# Health related quality of life and healthcare utilization among adults with diabetes and kidney and eye complications in the United States

**DOI:** 10.1186/s12955-020-01336-w

**Published:** 2020-03-30

**Authors:** Abdulkarim M. Meraya, Monira Alwhaibi

**Affiliations:** 1 Department of Clinical Pharmacy, College of Pharmacy, Jazan University, P.O Box 114-45124, Jazan, Saudi Arabia; 2 Pharmacy Practice Research Unit, College of Pharmacy, Jazan University, Jazan, Saudi Arabia; 3 Department of Clinical Pharmacy, College of Pharmacy, King Saud University, Riyadh, Saudi Arabia; 4 Medication Safety Research Chair, College of Pharmacy, King Saud University, Riyadh, Saudi Arabia

**Keywords:** Health-related quality of life, Chronic kidney disease, Eye complications, Healthcare utilization, Diabetes

## Abstract

**Background:**

One-third of adults with diabetes in the United States have chronic kidney disease (CKD), and 19% of them have eye complications (ECs). However, little is known about the Health-related Quality of Life (HRQoL) of adults with both of these diabetes-related complications. Therefore, the purpose of this study is to examine differences in the HRQoL, mental health, and healthcare utilization of adults with diabetes who have CKD, ECs, both or neither.

**Methods:**

A cross-sectional study design was implemented using data from multiple panels (2009–2015) of the Medical Expenditure Panel Survey. HRQoL was measured using the SF-12 Physical and Mental Component Summary (PCS & MCS) scores. The HRQoL, mental health, and healthcare utilization of four mutually exclusive groups: 1) diabetes with both CKD and ECs; 2) diabetes with CKD only; 3) diabetes with ECs only, and 4) diabetes with neither CKD nor ECs were compared. In all analyses, adults with neither CKD nor ECs were the reference group.

**Results:**

There were 8415 adults with diabetes who met the inclusion criteria. Approximately, 75% of the study sample had neither CKD nor ECs, 13.3% had ECs only, 5.7% had CKD only, and 5.5% had both CKD and ECs. In the adjusted analyses, adults with both CKD and/or ECs complications exhibited significantly lower HRQoL compared to those with neither CKD nor ECs. Mental illness and psychological distress were higher among adults with both CKD and ECs compared to those with neither CKD nor ECs. Furthermore, adults with CKD and/or ECs had higher polypharmacy, inpatient and emergency services use compared to those with neither CKD nor ECs.

**Conclusions:**

The results indicate that the presence of both CKD and/or ECs was negatively associated with poor HRQoL, poor mental health, higher psychological distress and healthcare utilization in adults with diabetes. The findings emphasize the need for routine assessment and treatment for diabetes-related CKD and/or ECs complications to improve the quality of care for individuals with diabetes.

## Background

Diabetes is a highly prevalent chronic condition. An estimated 381.8 million had diabetes in 2013, and it is projected to rise to 591.8 million in 2035, worldwide [1]. In the United States (US), nearly 30.2 million adults were diagnosed with diabetes, of which one third had chronic kidney disease (CKD) [2], and 19% had eye complications (ECs) [3]. Diabetes-related CKD and/or ECs can worsen the health outcomes and can potentially increase diabetes-related morbidity and mortality. For instance, adults with diabetes and related CKD and/or ECs complications are more prone to die prematurely, and the risk of death is exponential to the severity of the diseases [3]. Globally, the healthcare expenditures on diabetes are projected to exceed US$ 490 billion in 2030 [4], and a large proportion of costs spent on treating the complications of diabetes [5]. Diabetes-related CKD and/or ECs are associated with high healthcare expenditures and utilization [6, 7]. Diabetes-related complications were clearly associated with hospitalization; the estimated proportion of diabetic adults who were hospitalized is 20% higher for persons reporting ≥3 complications as compared to diabetic adults with no complications [6]. Moreover, the number of diabetes-related complications has resulted in extra hospital visits and healthcare cost [7].

Further, CKD and/or ECs complications are associated with lower health-related quality of life (HRQoL) [8–12]. HRQoL is a multi-dimensional concept that includes aspects of overall quality of life that can affect patient’s health—either physical or mental functioning, according to the Centers for Disease Control and Prevention (CDC) definition [13]. It goes beyond direct measures of population health, life expectancy, and causes of death, and focuses on the impact health status has on quality of life. Previous studies have indicated that adults with CKD experience lower mental and physical health than the general public [10, 11, 14] or individuals with diabetes [15]. Also, adults with ECs including those with diabetic retinopathy have lower HRQoL than those without ECs [15].

Diabetes is also associated with a high risk of hospitalization [16], and emergency department use [17, 18]. Adults with diabetes may also have multiple co-existing conditions [18–20]. As a result, diabetes is associated with high rates of polypharmacy (treatment with multiple medications concurrently) [21, 22]. Diabetes-related complications can further increase the risk of healthcare utilization.

The above-mentioned studies indicate the high humanistic and financial burden of diabetes-related CKD and eye complications, therefore more understanding is needed. The relationship between CKD or ECs and HRQoL has been well documented; however, no study has yet examined the effect of the combination of CKD and ECs on HRQoL among adults with diabetes in the US. Besides, little is known about the difference in healthcare utilization among adults with diabetes who have different types of complications in the US [17, 18, 22]. Therefore, the current study has two objectives: 1. Primary objective – to examine the differences in the HRQoL, and mental health of adults with diabetes who have CKD, ECs, both or neither, and 2. Secondary objective - to estimate the differences in the likelihood of healthcare utilization (i.e., inpatient services, outpatient services, and emergency department use) among the aforementioned groups.

## Methods

### Study design

The cross-sectional study design was employed using data from multiple panels (2009–2015) of the Medical Expenditure Panel Survey (MEPS).

### Data source

Households, diabetes care surveys, prescription medications, and medical conditions files from the MEPS were used in this study [23]. The MEPS is an annual household survey of the US non-institutionalized civilian population [23]. Four years of data (2009, 2011, 2013, and 2015) were combined to increase the sample size. Based on the recommendations of the Agency for Healthcare Research and Quality, the current study used alternate years to avoid duplicate observations of the same participant [24].

In MEPS, information regarding surveyed participants’ mental and physical health, demographic and socioeconomic characteristics, employment, access to care, and satisfaction with health care is available in the household component of the survey. Medical conditions reported by the participants were extracted from the household file or the medical conditions file. The MEPS prescription medication file provides information about the therapeutic classes of medicines in connection with the Multum Lexicon database [23].

### Study sample

The study sample consists solely of adults, aged 21 years or older, with diabetes. In the MEPS, individuals with diabetes are those who responded positively to the question, “Have you ever been told by a doctor or health professional that you have diabetes?” These individuals were then sent a diabetes care survey to gather information related to diabetes management, diabetes-related complications, and recommended preventive care.

### Measures

#### Outcomes

##### Health-related quality of life

HRQoL in MEPS data was assessed via the self-administered questionnaire to adults aged 18 years old or older participating in the MEPS. HRQoL includes the physical and mental health domains, Physical Component Summary (PCS) and Mental Component Summary (MCS). For physical health, MEPS provides information on the physical component summary (PCS) scores derived from the 12-item short-form health survey (SF-12). The PCS encompasses physical functioning, role-physical, bodily pain, and general health. The PCS scores range from 0 to 100, with higher scores representing better self-reported physical health [25]. For mental health, MEPS also provides information on the mental component summary (MCS) scores derived from the SF-12. The MCS includes social functioning, role emotional, mental health and vitality. The MCS scores also range from 0 to 100, with higher scores representing better self-reported mental health [25]. It must be noted that the domains and items in the PCS have some weight in the MCS, and vice versa.

##### Mental illness

For the purposes of this study, mental illness is determined to be present if participants indicated a diagnosis of depression or anxiety. Psychological distress: Psychological distress was measured using the six-item Kessler-6 Non-Specific Psychological Distress Scale (K6), [26], which includes the following questions: “In the past 30 days, about how often did you feel: (1) so sad nothing could cheer you up? (2) nervous? (3) restless or fidgety? (4) hopeless? (5) that everything was an effort? (6) worthless?” The responses to these six questions were rated on a five-point scale. In the present study, the summary score from the K6 scale is used to measure psychological distress. The summary score from the K6 scale ranges from 0 to 24, and a score of 13 or greater is considered to reflect serious psychological distress, which is a strong indicator of mental illness [27, 28]. Therefore, adults in this study are either classified as those with serious psychological distress (13 or greater) or those without serious psychological distress (less than 13).

##### Healthcare utilization

Polypharmacy: The study sample was divided into two groups: adults with polypharmacy (≥6 medication classes) and adults without polypharmacy (< 6 medication classes), in accordance with previous studies [29, 30].

Medical services: Healthcare utilization includes the use of services related to (inpatient, outpatient, and emergency department (ED)). Adults in the study sample were categorized based on whether or not they had used medical services (inpatient services (yes, no); outpatient services (yes, no); ED services (yes, no)) [23].

#### Key explanatory variable

In this study, adults with diabetes were classified into four mutually exclusive groups: 1) diabetes with both CKD and ECs; 2) diabetes with CKD only; 3) diabetes with ECs only, and 4) diabetes with neither CKD nor ECs. CKD was identified by a positive response to the question, “Has your diabetes caused problems with your kidneys?” Meanwhile, eye complications (ECs) were identified by a positive response to the question, “Has your diabetes caused problems with your eyes that needed to be treated by an ophthalmologist?”

#### Other explanatory variables

The selection of the independent variables was guided by the Andersen’s Expanded Behavioral Model [31]. Based on this model, health outcomes and healthcare use may be influenced by predisposing factors (e.g., age and sex), enabling factors (e.g., education level and health insurance), need factors (e.g., physical and mental health) and personal health practices (e.g., smoking status). Also, previously conducted studies have shown that demographics, socioeconomic status, health insurance and comorbid conditions are associated with HRQoL [32, 33]. Other explanatory variables include sex (female, male); age (21–39, 40–49, 50–64 and 65 years or older); race/ethnicity (White, African American, Latino, and other); marital status (married, separated/divorced, widowed and never married); poverty status: poor (less than 100% federal poverty line), near poor (100% to less than 200%), middle income (200% to less than 400%), and high income (greater than or equal to 400%); health insurance coverage (private, public, uninsured); prescription drug coverage (yes or no); presence of other co-occurring physical conditions (asthma, arthritis, cancer, gastroesophageal reflux disease (GERD), heart diseases, hypertension, osteoporosis, thyroid, and chronic obstructive pulmonary disease (COPD)); smoking status (current smoker and others); Body Mass Index (BMI) categories (underweight/normal (≤24.9), overweight (25–29.9), obese (≥ 30)); and physical activity (vigorous or moderate activities at least 3 days a week and other).

### Statistical techniques

Chi-square tests were employed to examine the associations between the studied groups and other explanatory variables in the bivariate analysis. ANOVA tests were used to assess the difference in the means of MCS and PCS between the four diabetes groups. Multivariable ordinary least squares (OLS) regressions were used to examine the associations between the groups and the MCS and PCS, separately. Domains and items in the PCS have some weight in the MCS, and vice versa. Therefore, we did not include MCS as an explanatory variable in the model where PCS was an outcome and vice versa to address the endogeneity due to bi-directional relationships. However, we have conducted sensitivity analyses to test the difference between a model adjusted for MCS and a model that did not adjust for it. Multivariable logistic regressions were used to assess the relationships between the studied groups and the presence of mental illness, psychological distress, polypharmacy, inpatient services use, outpatient services use, and ED use, separately. In all of the regression models, the reference group was “adults with neither CKD nor ECs”. In all adjusted models, the following variables were controlled for sex, age, race/ethnicity, marital status, poverty status, health insurance coverage, prescription drug coverage, the number of co-occurring physical and mental health conditions, smoking status, BMI, and physical activity. All analyses were conducted using survey procedures in the Statistical Analysis System (SAS®) version 9.4 and STATA 15.1. Diabetes care survey weights were used in the analyses. These weights adjust for diabetes care surveys nonresponse and weights to the number of individuals with diabetes in the U.S. civilian non-institutionalized population in a given year.

## Results

### Description of the study sample

Table 1 displays the characteristics of the studied adults in all four groups. The study sample consists of 8415 adults with diabetes, of which 75.5% had neither CKD nor ECs, 13.3% had ECs only, 5.7% had CKD only, and 5.5% had both CKD and ECs. Age, race, poverty status, health insurance, prescription drug coverage, the co-occurrence of chronic physical conditions, co-occurrence of chronic mental conditions, BMI, and smoking status were found to be significantly associated with the presence of CKD or ECs in the bivariate analysis. For example, a higher percentage of African Americans than white adults had both CKD and ECs (6.9% vs 4.9%). Similarly, a higher percentage of adults with a low economic status had both CKD and ECs compared to those with higher income. Moreover, the presence of the two diabetes-related complications was more prevalent among the participants with more than five physical chronic conditions than those with no co-occurring physical chronic conditions (9.8% vs 2.2%).

**Table 1:** Characteristics of the study sample by presence of diabetes-related complications, adults with diabetes, weighted row percentages. Medical Expenditure Panel Survey, panels 2009–2015

	Total	Neither CKD nor ECs	Eye Complications	CKD	CKD & Eye Complications	***P***-Value
	Wt. (%)	Wt. (%)	Wt. (%)	Wt. (%)	Wt. (%)	
**ALL**		75.5	13.3	5.7	5.5	
**Sex**						0.297
Female	50.7	74.5	14.0	5.7	5.8	
Male	49.3	76.6	12.6	5.6	5.2	
**Age**						0.030
21–39	6.5	78.6	13.3	5.1	3.1	
40–49	12.9	79.0	12.0	4.0	5.0	
50–64	37.9	76.2	13.2	5.5	5.1	
65, +	42.7	73.4	14.0	6.4	6.3	
**Race**						< 0.001
White	61.3	77.3	11.4	6.4	4.9	
African American	15.4	70.8	17.3	5.1	6.9	
Latino	15.2	71.7	17.9	3.8	6.6	
Other	8.1	78.0	12.6	4.7	4.8	
**Marital status**						0.053
Married	57.4	77.1	12.4	5.4	5.1	
Separated/Divorced	18.1	73.7	14.4	5.7	6.2	
Widow	13.7	71.6	15.1	6.7	6.7	
Never Married	10.8	75.0	14.3	6.0	4.7	
**Poverty status**						< 0.001
Poor	14.4	67.5	17.8	7.1	7.6	
Near Poor	22.2	71.2	15.9	5.9	7.0	
Middle Income	31.0	75.7	13.3	5.9	5.1	
High Income	32.4	81.8	9.7	4.7	3.8	
**Health insurance**						< 0.001
Private	58.2	79.8	11.3	4.9	4.0	
Public	34.4	68.2	16.8	7.0	8.1	
Uninsured	7.5	75.9	13.9	5.2	5.0	
**Prescription Drug coverage**						< 0.001
Yes	92.2	74.7	13.7	5.9	5.8	
No	7.8	85.5	9.8	3.2	1.5	
**Chronic Physical Conditions Number**						< 0.001
No Physical condition	11.8	84.7	10.2	2.9	2.2	
1–2	48.7	78.6	13.0	4.2	4.3	
3–4	30.8	71.0	14.4	7.3	7.3	
= > 5	8.7	62.1	16.0	12.1	9.8	
**Chronic Mental Conditions Number**						
No mental condition	86.2	76.4	13.1	5.5	5.0	< 0.001
> = 1	13.8	70.2	14.9	6.4	8.4	
**Body mass index**						
Underweight /Normal	14.1	73.9	16.5	3.5	6.2	0.023
Overweight	29.8	74.8	13.5	6.4	5.3	
Obese	54.6	76.5	12.4	5.8	5.4	
**Smoking status**						< 0.001
Current smoker	14.6	73.6	15.6	5.5	5.4	
Other	85.4	76.0	13.0	5.6	5.4	

Abbreviations: *CKD* Chronic Kidney Disease, *Wt.* Weighted

**Note:** Based on 8415 adults aged 21 years or older, alive during the calendar years, and reported having diabetes. The p- values were derived from the chi-square tests between groups of adults with complications and explanatory variables. Physical chronic conditions included asthma, arthritis, cancer, gastroesophageal reflux disease, heart diseases, hypertension, osteoporosis, thyroid, and Chronic obstructive Pulmonary disease. Mental chronic conditions included anxiety and/or depression

### HRQoL: physical health component

The mean PCS scores for the four groups are: diabetes with neither CKD nor ECs [42.4 (SE = 0.19)]; diabetes with ECs only [37.6 (SE = 0.5)]; diabetes with CKD only [35.1 (SE = 0.8)]; and diabetes with both CKD and ECs [32.4 (SE = 0.7)]. There was a significant difference in HRQoL (PCS) between the four diabetes groups (Table 2). For example, adults with both CKD and/or ECs exhibit significantly lower PCS scores than adults without CKD or ECs in the unadjusted OLS regressions. After controlling for sex, age, race/ethnicity, marital status, poverty status, health insurance coverage, prescription drug coverage, number of mental and physical health conditions, BMI, smoking status and physical activity, a significantly lower PCS scores was observed among adults with ECs only (β = − 2.99, *p* < 0.001), with CKD only (β = − 3.78, *p* < 0.001), and with both CKD and ECs (β = − 6.16, *p* < 0.001) compared to those with neither CKD nor ECs. In the sensitivity analyses, we have adjusted for all other explanatory variables and MCS in one model where PCS was an outcome and conducted another model in which we adjusted only for all other explanatory variables. We found that there is no difference in the results between a model adjusted for MCS and model did not adjust for MCS. Therefore, we believe that removing MCS from the model did not lead to omitted variable bias. The results are shown in Table 4 in Appendix.

**Table 2:** Parameter estimates and standard errors of diabetes-related complications categories from OLS regression. Outcomes: MCS and PCS scores, adults with diabetes. Medical Expenditure Panel Survey, panels 2009–2015

	MCS			PCS		
Explanatory Variable	Beta	SE	Significance	Beta	SE	Significance
	**Unadjusted Model**					
Eye Complications	−3.49	0.47	< 0.001	−4.76	0.52	< 0.001
CKD	−3.54	0.69	< 0.001	−7.28	0.79	< 0.001
CKD & Eye Complications	−5.27	1.81	< 0.001	−9.94	0.72	< 0.001
Neither CKD nor ECs	*Reference Group*			*Reference Group*		
	**Adjusted Model**					
Eye Complications	−2.29	0.40	< 0.001	−2.99	0.41	< 0.001
CKD	−2.49	0.65	< 0.001	−3.78	0.64	< 0.001
CKD & Eye Complications	−3.16	0.71	< 0.001	−6.16	0.62	< 0.001
Neither CKD nor ECs	*Reference Group*			*Reference Group*		

Abbreviations: *SE* Standard error, *CKD* Chronic Kidney Disease, *PCS* Physical Component Summary, *MCS* Mental Component Summary

**Note:** Based on 8415 adults aged 21 years or older, alive during the calendar years, and reported having diabetes

PCS adjusted model included sex, age, race/ethnicity, marital status, poverty status, health insurance coverage, number of mental and physical health conditions, BMI, smoking status, geographic area of residence and physical activity

MCS adjusted model included sex, age, race/ethnicity, marital status, poverty status, health insurance coverage, number of physical health conditions, BMI, smoking status, geographic area of residence and physical activity

### HRQoL: mental health component

The mean MCS scores for the four groups are: diabetes with neither CKD nor ECs [50.8 (SE = 0.19)]; diabetes with ECs only [47.3 (SE = 0.44)]; diabetes with CKD only [47.3 (SE = 0.68)]; and diabetes with both CKD and ECs [45.6 (SE = 0.80)]. In the unadjusted OLS model, adults with both CKD and/or ECs exhibit significantly lower MCS scores than those with neither CKD nor ECs (Table 2). After controlling for sex, age, race/ethnicity, marital status, poverty status, health insurance coverage, prescription drug coverage, number of physical health conditions, BMI, smoking status and physical activity, a significantly lower MCS scores was observed among adults with CKD and/or ECs compared to those with neither CKD nor ECs.

### Other mental health measures

#### Mental illness

Using mental illness and serious psychological distress as outcomes, Table 3 displays the adjusted odds ratios and the standard error for the four studied groups. Adults with both CKD and ECs were more likely to have a mental illness (OR = 1.42; *P* = 0.043) than those with neither CKD nor ECs after controlling for sex, age, race, marital status, poverty status, health insurance coverage, prescription drug coverage, number of physical health conditions, BMI, smoking status and physical activity. Nevertheless, concerning having a mental illness, no differences were found between adults with either CKD or ECs only and those with neither CKD nor ECs.

**Table 3:** Unadjusted and adjusted odds ratio and 95% confidence intervals for diabetes-related complications croups from logistic regressions

	Wt.%(95%CI)	Unadjusted		Adjusted	
		OR [95% CI]	Significance	AOR [95% CI]	Significance
**Mental Illness**					
Eye Complications	15.4 (12.7–18.5)	1.23 [0.96, 1.59]	0.093	1.14 [0.87, 1.48]	0.324
CKD	15.7 (11.9–20.3)	1.26 [0.91, 1.33]	0.16	0.96 [0.68, 1.36]	0.827
CKD & Eye Complications	21.3 (16.6–26.8)	1.84 [1.33, 2.53]	< 0.001	1.42 [1.01, 2.01]	0.043
Neither CKD nor ECs	12.8 (11.7–14.0)	*Reference*			
**Serious Psychological Distress**					
Eye Complications	13.7 (11.2–16.7)	2.12 [1.62, 2.78]	< 0.001	1.67 [1.26, 2.21]	< 0.001
CKD	12.8 (9.4–17.3)	1.97 [1.36, 2.78]	< 0.001	1.39 [0.92, 2.74]	0.119
CKD & Eye Complications	17.9 (13.6–23.2)	2.92 [2.07, 4.11]	< 0.001	1.92 [1.35, 2.74]	< 0.001
Neither CKD nor ECs	7.0 (6.1–7.9)	*Reference*			
**Polypharmacy Use**					
Eye Complications	57.2 (53.6–60.7)	1.47 [1.27, 1.72]	< 0.001	1.27 [1.07, 1.51]	0.006
CKD	73.7 (68.7–78.1)	3.09 [2.45, 3.92]	< 0.001	2.25 [1.66, 3.05]	< 0.001
CKD & Eye Complications	74.8 (70.2–78.8)	3.27 [2.59, 4.15]	< 0.001	2.09 [1.57, 2.79]	< 0.001
Neither CKD nor ECs	47.5 (45.8–49.1)	*Reference*			
**Inpatient Services Use**					
Eye Complications	21.0 (18.1–24.3)	1.73 [1.41, 2.12]	< 0.001	1.49 [1.20, 1.85]	< 0.001
CKD	26.9 (22.3–32.2)	2.39 [1.82, 3.14]	< 0.001	1.87 [1.41, 2.50]	< 0.001
CKD & Eye Complications	31.9 (26.7–37.5)	3.03 [2.33, 3.94]	< 0.001	2.22 [1.67, 2.95]	< 0.001
Neither CKD nor ECs	13.4 (12.3–14.4)	*Reference*			
**Outpatient Services Use**					
Eye Complications	95.7 (94.4–96.8)	1.48 [1.07, 2.04]	0.015	1.35 [0.92, 1.98]	0.116
CKD	95.7 (91.0–98.0)	1.48 [0.66, 3.35]	0.339	0.78 [0.34, 1.79]	0.560
CKD & Eye Complications	96.9 (94.1–98.3)	2.03 [1.03, 4.02]	0.041	1.12 [0.54, 2.69]	0.638
Neither CKD nor ECs	93.8 (92.9–94.6)	*Reference*			
**Emergency Department Services Use**					
Eye Complications	30.3 (27.1–33.7)	1.86 [1.55, 2.24]	< 0.001	1.63 [1.33, 1.98]	< 0.001
CKD	36.0 (30.8–41.6)	2.42 [1.86, 3.13]	< 0.001	1.92 [1.50, 2.47]	< 0.001
CKD & Eye Complications	35.8 (30.7–41.3)	2.39 [1.89, 3.03]	< 0.001	1.77 [1.38, 2.28]	< 0.001
Neither CKD nor ECs	18.9 (17.6–20.2)	*Reference*			

Abbreviations: *Wt.* Weighted, *CI* Confidence Interval, *AOR* Adjusted Odds Ratio, *CKD* Chronic Kidney Disease, *OR* Odds Ratio

**Note:** Based on 8415 adults aged 21 years or older, alive during the calendar years, and reported having diabetes

**Mental Health adjusted models** included sex, age, race/ethnicity, marital status, poverty status, health insurance coverage, number of physical health conditions, BMI, smoking status, geographic area of residence and physical activity

**Healthcare Utilization adjusted model**s included sex, age, race/ethnicity, marital status, poverty status, health insurance coverage, drug prescription coverage, number of mental and physical health conditions, BMI, smoking status, geographic area of residence and physical activity

#### Serious psychological distress

Similarly, adults with both CKD and ECs were more likely to experience serious psychological distress (OR = 1.92; *P* < 0.001) than those with neither CKD nor ECs in the adjusted analysis. However, with experiencing serious psychological distress, no significant differences were found between adults with CKD compared to those with neither CKD nor ECs.

### Healthcare utilization

#### Polypharmacy

After controlling for sex, age, race/ethnicity, marital status, poverty status, health insurance coverage, drug prescription coverage, number of mental and physical health conditions, BMI, smoking status and physical activity, adults with ECs only (OR = 1.27; *p* = 0.006), with CKD only (OR = 2.25; *p* < 0.001), with both CKD and ECs (OR = 2.09; *p* < 0.001), were significantly more likely to have polypharmacy than those with neither CKD nor ECs.

#### Medical services

As displayed in Table 3, a higher proportion of adults with CKD only had inpatient and emergency department services use than those with ECs*.* In the adjusted analyses, adults with ECs only (OR = 1.49; *p* < 0.001), those with CKD only (OR = 1.87; *p* < 0.001), and those with both CKD and ECs (OR = 2.22; *p* < 0.001) were significantly more likely to use inpatient services than those with neither CKD nor ECs. For outpatient services use, there were no differences exist between the four groups. However, adults with both CKD and/or ECs were more likely to use emergency department services than those with neither CKD nor ECs.

## Discussion

The current study examines the differences in HRQoL, mental health, and healthcare utilization among adults with diabetes who have CKD, ECs, both or neither. The study found that adults with both CKD and/or ECs reported significantly lower HRQoL, poor mental health, higher psychological distress and healthcare utilization than those with neither CKD nor ECs.

Adults with diabetes and CKD and/or ECs reported significantly lower HQRoL compared to those with neither CKD nor ECs. Our results have shown linear relationship between number of diabetes-related complications and mental and physical domains of HRQoL. Adults with ECs only and CKD only exhibited 2.99 and 3.78 lower scores on PCS as compared to those with neither of them. However, adults with both CKD and ECs exhibited 6.2 lower scores on PCS. Likewise, adults with both CKD and ECs exhibited a greater declining on MCS. These findings suggest that HRQoL worsened as the number of diabetes-related complications increased in adults with diabetes. Future research is required to examine the relationships between different diabetes-related complications and health measures to better customize health programs and initiatives. Mental health also differed among the studied groups. Adults with CKD and/or ECs were more likely to experience psychological distress than those with neither condition. This finding points out the effects of CKD and/or ECs not only on HRQoL but also on the mental illness and psychological distress.

The results of this study also indicated higher polypharmacy among adults with ECs and/or CKD, who were more likely to take six or more prescription drugs of different classes concurrently. This is not surprising as the number of comorbidity increases, individuals are usually at a high risk of polypharmacy use. It has to be noted that the use of polypharmacy can affect the health outcomes of adults with diabetes. For example, Jesus et al. stated that individuals with diabetes and associated with comorbidity such as CKD who were undergoing expensive therapies experience physical and mental distress and have significantly lower levels of satisfaction in their lives [34]. Polypharmacy might increase the cost of therapy and could be another contributing factor in lowering HRQoL in such individuals. However, it is important to note that CKD is a complex disease, and it is associated with several problems at the biological level [35], such as high blood pressure, anemia, weak bones, poor nutritional health, and nerve damage [35]. As a result, the management of CKD often requires multiple prescription drugs, including analgesics, anti-hypertensive agents, and dietary supplements.

Moreover, the results of the current study revealed that adults with CKD and/or ECs are more likely to use inpatient and emergency department services than those with neither condition. It has to be noted that inpatient and emergency department services are associated with high healthcare expenditure [36, 37]. This may explain why adults with diabetes-related complications exhibit higher healthcare expenditures than those without such complications [38]. Our analyses also indicated that adults with CKD only had high healthcare utilization. Additionally, published studies also documented higher healthcare utilization among adults with CKD [39]. Therefore, it seems that CKD is the main driver for more healthcare utilization among adults with diabetes in the US. Further research is needed to identify the factors associated with hospitalization and ED visits among adults with CKD to decrease healthcare expenditure. Furthermore, to prevent hospitalization and ED visits among adults with both ECs and CKD, prevention strategies may be needed to minimize diabetes-related complications. Further research is needed to identify the factors associated with hospitalization among adults with CKD to decrease healthcare expenditure.

### Practical implications

Many practical implications can emanate from the current study findings. As findings of this study suggest that adults with CKD and/or ECs have poorer HRQoL and higher healthcare utilization; thus, interventions and health programs are crucial to improve HRQoL and reduce healthcare utilization among adults with diabetes-related complications. Initiatives and educational programs are required to address high blood sugar and other lifestyle modifications (including weight reduction, physical activities, and diet) to mitigate the effects of CKD and ECs on HRQoL and healthcare utilization among adults with diabetes. It has been documented that participation in diabetes self-management activities, including general diet and exercise, are associated with higher HRQoL among adults with diabetes and with or without CKD [40].

Besides, published researches have indicated that diabetes education was associated with higher HRQoL [41–43]. Therefore, the present findings also possess implications for healthcare professionals and educators, who often coordinate care for individuals with diabetes-related complications. For example, healthcare professionals need to address diabetes-related complications to improve diabetes health outcomes. As adults with diabetes-related complications are at high risk of using multiple medications, healthcare providers should monitor closely for pharmacokinetics of drugs, drug-drug and drug-disease interactions. Further, as chronic diabetes-related complications can be devastating, therefore prevention interventions are important. An effective preventive measure to control glucose levels can reduce the risk and progression of diabetes-related complications [44], and can reduce the healthcare cost and utilization [45].

### Strengths and limitations

The current study has both strengths and limitations. To the best of the authors’ knowledge, this is one of the few studies to examine the humanistic (i.e., HRQoL) and economic burden of kidney and eye complications among adults with diabetes. A representative sample of U.S. adults with diabetes was used, and a comprehensive list of confounders was included in the analyses. However the study has some limitations. As all data was self-reported, it may be subject to recall bias. Furthermore, information regarding the severity of participants’ diabetes, ECs, and CKD was not available, and therefore, they were not considered in the current study. In the analyses, we did not differentiate between Type 1 and Type 2 diabetes, as this information is not available in the MEPS. Besides, information on other diabetes-related complications such as neuropathy is not available in the MEPS. In addition, MEPS does not provide information on individuals with undiagnosed diabetes. Thus, we restricted our analyses to individuals who were diagnosed with diabetes by a healthcare professional. Finally, since this is a cross-sectional study, it was not possible to determine the temporal relationships between the studied factors and the outcomes.

## Conclusion

In the present study, the results indicate that the presence of both CKD and ECs was negatively associated with poor HRQoL (physical health domain), poor mental health, higher psychological distress and healthcare utilization as compared to those without CKD and ECs This finding supports the need for comprehensive care for diabetic adults with diabetes-related complications.

## Data Availability

The dataset supporting the conclusions of this article is available from the Medical Expenditure Panel Survey (MEPS) database, and openly made available for researchers at the following website: https://meps.ahrq.gov/data_stats/download_data_files.jsp.

## Appendix

**Table 4:** Parameter estimates and standard errors of diabetes-related complications categories from OLS regression, adults with diabetes. Medical Expenditure Panel Survey, panels 2009–2015

	Outcome: PCS. Adjusted for MCS and other factors			Outcome: PCS. Adjusted for other factors but not MCS		
	Beta	SE	Significance	Beta	SE	Significance
Eye Complications	−2.93	0.42	< 0.001	−2.99	0.41	< 0.001
CKD	−3.68	0.64	< 0.001	−3.78	0.64	< 0.001
CKD & Eye Complications	−6.04	0.62	< 0.001	−6.16	0.62	< 0.001
Neither CKD nor ECs	*Reference Group*			*Reference Group*		

Abbreviations: *SE* Standard error, *CKD* Chronic Kidney Disease, *PCS* Physical Component Summary, *MCS* Mental Component Summary

**Note:** Based on 8415 adults aged 21 years or older, alive during the calendar years, and reported having diabetes

**PCS** adjusted model included sex, age, race/ethnicity, marital status, poverty status, health insurance coverage, number of mental and physical health conditions, BMI, smoking status, geographic area of residence and physical activity
